# Controlled formation of gold nanoparticles with tunable plasmonic properties in tellurite glass

**DOI:** 10.1038/s41377-023-01324-x

**Published:** 2023-12-07

**Authors:** Yunle Wei, Jiangbo Zhao, Sindy Fuhrmann, Roman Sajzew, Lothar Wondraczek, Heike Ebendorff-Heidepriem

**Affiliations:** 1 Institute for Photonics and Advanced Sensing, School of Physics, Chemistry and Earth Sciences, The University of Adelaide, Adelaide, SA 5005 Australia; 2 School of Engineering, University of Hull, Hull, HU6 7RX UK; 3 Institute of Glass Science and Technology, TU Bergakademie Freiberg, 09599 Freiberg, Germany; 4 Otto Schott Institute of Materials Research, University of Jena, 07743 Jena, Germany; 5 Leibniz Institute of Photonic Technology, 07745 Jena, Germany; 6 Center of Energy and Environmental Chemistry, University of Jena, 07743 Jena, Germany

**Keywords:** Nanoparticles, Nanophotonics and plasmonics

## Abstract

Silicate glasses with metallic nanoparticles (NPs) have been of intense interest in art, science and technology as the plasmonic properties of these NPs equip glass with light modulation capability. The so-called striking technique has enabled precise control of the in situ formation of metallic NPs in silicate glasses for applications from coloured glasses to photonic devices. Since tellurite glasses exhibit the unique combination of comparably easy fabrication, low phonon energy, wide transmission window and high solubility of luminescent rare earth ions, there has been a significant amount of work over the past two decades to adapt the striking technique to form gold or silver NPs in tellurite glasses. Despite this effort, the striking technique has remained insufficient for tellurite glasses to form metal NPs suitable for photonic applications. Here, we first uncover the challenges of the traditional striking technique to create gold NPs in tellurite glass. Then, we demonstrate precise control of the size and concentration of gold NPs in tellurite glass by developing new approaches to both steps of the striking technique: a controlled gold crucible corrosion technique to incorporate gold ions in tellurite glass and a glass powder reheating technique to subsequently transform the gold ions to gold NPs. Using the Mie theory, the size, size distribution and concentration of the gold NPs formed in tellurite glass are determined from the plasmonic properties of the NPs. This fundamental research provides guidance for designing and manipulating the plasmonic properties in tellurite glass for photonics research and applications.

## Abstract (web summary)

Tunable plasmonic features were achieved in tellurite glass via new ways to control the in situ formation of gold nanoparticles, enabling investigation and utilising plasmonic properties for photonics research and applications.

## Graphical abstract

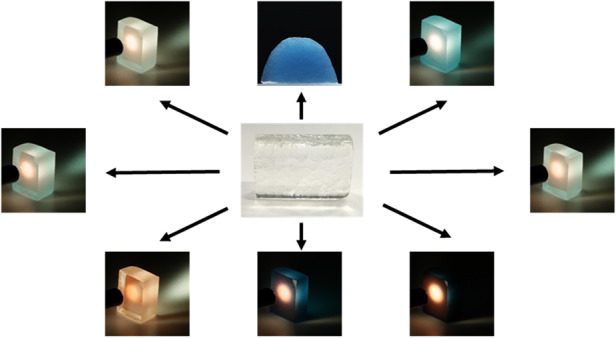

## Introduction

The fascination of silicate glasses containing metallic nanoparticles (NPs, e.g., gold Au or silver Ag) in decoration and art has lasted for centuries (if not millennia). Because of their applications as optical elements (such as polarisers)^1^ as well as the roles they are expected to play in applications such as optoelectronics, photonics, sensing, electrochemistry and biomedicine^2^, they imply a technological relevance and thus huge scientific interest. The intriguing functionalities of silicate glasses with Au or Ag NPs are intimately related to their unique optical properties originating from the localised surface plasmon resonance (LSPR). It is now well understood that the LSPR features—the spectral shape, position and intensity—are determined by the metallic NP properties (composition, size, shape and number density) as well as the refractive index of their surrounding medium^3^. Therefore, tailoring LSPR performance requires precise control of the metallic NP properties in a glass matrix through glass composition and manufacturing processes.

The LSPR features of Au and Ag NPs in silicate glasses can be well controlled by the well-established traditional striking technique (i.e., in situ formation of metallic NPs in glass matrix): (1) The glass batch is doped with Au and Ag compounds and in most cases also with polyvalent oxides (typically SnO/SnO_2_). (2) Melting the glass batch dissolves the Au and Ag compounds as colourless Au^+^ and Ag^+^ ions in the glass matrix—as proved by many indirect and direct experimental results in traditional oxide glasses such as silicate, germanate and borate as well as in geological silicate melts^4–6^. Quenching the melt yields the colourless precursor glass. (3) In the final colour striking step, reheating the precursor glass above the glass transition temperature induces the dissolved Au and Ag ions to be chemically reduced by the co-doped polyvalent ions to form metallic atoms that grow further into Au and Ag NPs that endow the entire glass with the desired colour and LSPR features^7,8^.

Over the past two decades, the striking technique has been applied to form Au and Ag NPs in a range of other glass systems to add LSPR-based functionality to these glass types^9–16^. Specifically, there has been a large amount of work to introduce Au or Ag NPs into tellurite glasses as they have a unique combination of properties: comparably easy fabrication (i.e., low melting temperature and high crystallisation stability), relatively good physical and chemical durability, low phonon energy (~800 cm^−1^), wide transmission window (0.4–5 µm) and high solubility of luminescent rare earth ions for hosting and guiding most of the rare earth fluorescence^17^. The tellurite glass type has been one of the most popular host matrix for studying the plasmonic effects of Ag or Au NPs on enhancing the fluorescence of rare earth dopants^18–22^. However, most of the published research only provided transmission electron microscopy (TEM) images of the Au/Ag doped tellurite glasses showing the formation of crystalline NPs^18–20^, with no further evidence confirming the elemental nature of the observed crystalline NPs. In addition, distinct LSPR features in tellurite glasses have been demonstrated in only a few reports^21,22^. The ability to tune the location and intensity of the LSPR band remains a challenge. However, this ability is critical for optimising the fluorescence enhancement of rare earth dopants^23^.

A decade ago, the unintentional formation of Au NPs in tellurite glass was discovered when developing the so-called melt doping technique to incorporate diamond nanocrystals in tellurite glasses^24^. In this technique, a batch of glass raw materials without NPs was melted in a gold crucible at a sufficiently high temperature (called melting temperature) to obtain a homogenised glass melt. Then, the temperature was lowered to a point (called doping temperature) that yields a glass viscosity which is sufficiently low to disperse the added diamond nanocrystals (and to cast the glass melt) while being sufficiently high to suppress the dissolution of the diamond nanocrystals in the glass melt^24^. Surprisingly, Au NPs were found in such a diamond nanocrystal doped tellurite glass, as evidenced by a distinct LSPR extinction band in the spectral range of 500–800 nm, causing the glass to exhibit distinct dichroic behaviour, namely blue colour in transmission and an orange colour in reflection^25^. The Au NP formation was proposed to occur as follows^25^: (1) a small amount of gold was dissolved into the hot tellurite glass melt in the form of gold ions at the glass melting temperature as a result of gold crucible corrosion; (2) the added diamond nanocrystals acted as reducing agents for the reduction of the gold ions into gold atoms, which further nucleated and grew into Au NPs. The formation of Au NPs via doping diamond nanocrystals into tellurite glass melts was found to be a stochastic process and hence unsuitable for controlled Au NP formation because of the complex chemistry between the diamond nanocrystals and the tellurite glass.

Here, we demonstrate the controlled in situ formation of Au NPs in tellurite glass via new ways to control the incorporation of Au^+^ ions into the glass melt and their chemical reduction to Au^0^ atoms. The incorporation of Au^+^ ions at targeted number densities is achieved through novel controlled gold crucible corrosion, while the controlled reduction of the Au^+^ ions to Au^0^ atoms is realised through a novel ‘striking’ technique utilising glass powder reheating. Compared with the traditional gold doping technique of adding gold salt to the glass batch, our technique of controlled gold crucible corrosion during tellurite glass melting enables comparably easy incorporation of high concentrations of Au^+^ ions in tellurite glass. The traditional Au NP formation technique of using polyvalent oxides (e.g., SnO/SnO_2_) as reducing agents for the formation of Au atoms is found to be unsuitable for tellurite glasses due to the chemical reduction of the Te^4+^ ions in the glasses to reduced tellurium species of lower oxidation state (Te^0^ and/or Te^2^^−^). By contrast, our technique of reheating glass powders free of polyvalent ions acting as reducing agents prevents reduced tellurium species formation while enabling the formation of Au^0^ atoms. By using first the controlled gold crucible corrosion technique to incorporate Au^+^ ions into the glass and then the glass powder reheating technique to chemically reduce Au^+^ to Au^0^, the concentration, size and size distribution of Au NPs in tellurite glass are tailored. This opens up, for the first time, tuning of both LSPR position and intensity in tellurite glass.

## Results

### Incorporation of Au^+^ ions into tellurite glass

As aforementioned in the “Introduction”, the first necessary condition for the formation of Au NPs is the incorporation of Au^+^ ions into the glasses that are used as so-called starting glasses for the second step of the Au NP formation. For silicate glasses, this is achieved by melting a glass batch with added gold salt. We tested the viability of this gold salt doping technique for the tellurite glass with a molar composition of 75TeO_2_–15ZnO-10Na_2_O (hereafter referred to as TZN). In addition, we developed a technique of incorporation of Au^+^ ions into glass via control of the corrosion of the gold crucible in which the tellurite glass is melted.

#### Gold salt doping technique

**Fig. 1:**
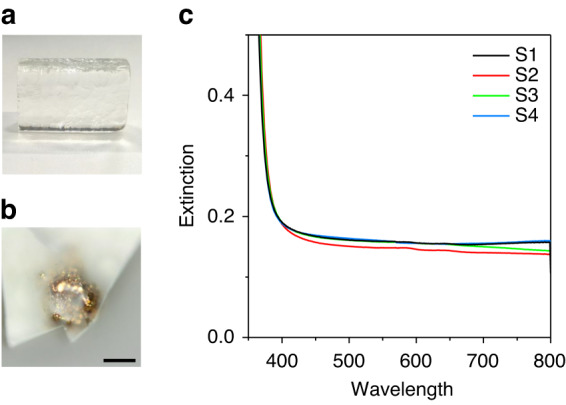
**a** Photograph of the TZN starting glass sample S1 doped with nominal 10 ppm Au in the form of HAuCl_4_. **b** Optical microscope image (bright field) of the Au metal particle observed in the residual glass at the bottom of the silica crucible used for the fabrication of S1. The scale bar is 100 µm. **c** Measured optical extinction spectra of 2 mm thick starting glass samples S1 (containing 3 ± 0.9 ppm Au), S2 (containing 6.3 ± 1.4 ppm Au), S3 (containing 19.1 ± 3.1 ppm Au) and S4 (containing 75.2 ± 3.5 ppm Au)

For testing the traditional approach of introducing Au^+^ ions into the glass matrix using the gold salt doping technique, we mixed 10 ppm Au (in the form of HAuCl_4_) into the glass batch, which was melted at 750 °C for 1 h in a silica crucible. The cast glass sample S1 is colourless (Fig. 1a), but a sub-mm sized Au metal particle was found in the residual glass at the bottom of the silica crucible (Fig. 1b), which indicates not all Au from the gold salt (HAuCl_4_) was incorporated as Au^+^ ions in the glass. Indeed, solution inductively coupled plasma mass spectrometry (ICP-MS) analysis confirms that the cast glass sample S1 contains only ~3 ppm of Au.

#### Controlled gold crucible corrosion technique

As described above, previous work found that melting of tellurite glass in a gold crucible led to the dissolution of Au into the tellurite glass melt in the form of Au^+^ ions^24^, which was undesired and thus subsequent work was dedicated to avoiding dissolution of Au into the tellurite glass melt^25,26^. By contrast, here we take advantage of this gold crucible corrosion by the tellurite glass melt as an intentional process to incorporate Au into tellurite glass in a controlled manner via varying the glass melting temperature. Three glass samples (S2, S3 and S4) were made by melting the undoped TZN batch in a gold crucible at 750, 800, and 850 °C (all for 1 h). These samples contain ~6, 19, and 75 ppm of Au, respectively, as measured by solution ICP-MS analysis (Table 1). This result indicates that the dissolution of Au via gold crucible corrosion increases with increasing glass melting temperature.

**Table 1:** Melting conditions and Au content for the TZN starting glass samples

Sample	Crucible	Batch doping	T_1_ (^o^C)	t_1_ (min)	Au content (ppm)
S1	silica	10 ppm Au	750	60	3 ± 0.9
S2	gold	none	750	60	6.3 ± 1.4
S3	gold	none	800	60	19.1 ± 3.1
S4	gold	none	850	60	75.2 ± 3.5
S5	silica	none	750	60	<0.1
S6	gold	2 w% SnO	750	60	n/m
S7	gold	2 w% SnO_2_	750	60	n/m
S8	gold	none	750	60	n/m

For S1, Au was doped into the glass batch in the form of HAuCl_4_ aqueous solution. Although S8 was fabricated under the same conditions as S2, a different sample name was used considering the starting glass of this sample was further powdered and mixed with ruby (Al_2_O_3_:Cr^3+^) nanocrystals for fabricating another sample (refer to the description in Table 2). ppm refers to parts per million by weight. n/m refers to not measured. The Au content in S1, S2, S3, S4 and S5 was measured via solution inductively coupled plasma mass spectrometry (ICP-MS), where <0.1 ppm refers to Au content below the detection limit (of 0.1 ppm)

As a control experiment, we made a glass sample S5 by melting an undoped TZN batch in a silica crucible at 750 °C for 1 h. As expected, no Au above the solution ICP-MS detection limit of 0.1 ppm was measured in this glass (Table 1).

#### Comparison of the mechanisms of both Au^+^ ion incorporation techniques

All four Au-containing glass samples, i.e., sample S1 made from gold salt doped batch melted in a silica crucible as well as the three samples S2, S3 and S4 made from undoped batches melted in gold crucibles are colourless (Fig. 1c). This indicates that all Au dissolved in the glasses via the gold salt doping technique (S1) or the controlled gold crucible corrosion technique (S2, S3, S4) is incorporated in ionic form in the glasses. This agrees with silicate glass doped with Au via the gold salt doping technique, where both Mossbauer and XAS (XANES/EXAFS) spectroscopy proved that Au exists as Au^+^ ions in colourless precursor silicate glass^5,27^.

For the same batch melting temperature and time (750 °C, 1 h), the gold salt doping technique leads to only ~3 ppm Au in the glass (S1), whereas the controlled gold crucible corrosion technique leads to a higher content of ~6 ppm Au (S2). The amount of dissolved Au via controlled gold crucible corrosion was further increased using higher melting temperature; up to 75 ppm Au for 850 °C (S4). These differences in Au^+^ concentrations in TZN glasses using the two Au^+^ incorporation techniques are attributed to two effects; (1) the critical step of the incorporation of ionic Au^+^ is the oxidation of metallic Au^0^, (2) the critical factor of the Au^0^ oxidation is the availability of oxygen.

For the gold salt doping technique, the amount of Au^+^ ions dissolved in the glass is the result of two competing processes; (1) thermal decomposition of gold salt to metallic gold at temperatures up to a maximum temperature (estimated to be ~750 °C and hereafter referred to as critical decomposition temperature), (2) oxidation of Au^0^ in the melt to Au^+^ at temperatures higher than the critical decomposition temperature. Pure HAuCl_4_ thermally decomposes to pure Au metal at 320 °C^28^. For a silicate glass batch doped with 200 ppm Au as aqua regia solution, thermal decomposition during heating up to the melting temperature (1400–1450 °C) led to the formation of Au NPs in the heated batch^7^. Based on these findings, we hypothesise that during heating up from room temperature to the critical decomposition temperature, the HAuCl_4_ in the glass batch is thermally decomposed to Au metal, resulting in the formation of Au particles by aggregation of the Au^0^ atoms. This behaviour shows that, for the gold salt doping technique, the critical step to incorporate Au in ionic form into TZN glass is the oxidation of Au^0^ (from the thermal decomposition of the gold salt) at temperatures above the critical decomposition temperature.

Similar to the gold salt doping technique, the controlled gold crucible corrosion technique relies on the oxidation of Au^0^, but the techniques differ in the origin of Au^0^; thermal decomposition of the gold salt for the former and the gold crucible itself for the latter. Furthermore, both techniques differ in the availability of oxygen for the Au^0^ oxidation and hence the amount of dissolved Au^+^ ions. For silicate melts, oxidation of Au^0^ in the melt to Au^+^ was found to increase with increasing melting temperature (at fixed oxygen partial pressure) and/or increasing oxygen partial pressure (at fixed temperature) of the melt^4,29^. In addition, the use of oxidising nitrate raw materials instead of carbonate in the glass batch is known to facilitate the oxidation of Au^0^ in silicate glass^7^. According to these results, we propose that for the gold salt doping technique, at melting temperatures higher than the critical decomposition temperature, the oxygen dissolved in the viscous glass leads to the oxidation of the Au metal particles. The observation of a micron-sized Au particle in the TZN glass despite the rather low gold salt concentration reveals the limited oxidation capacity of the glass melt, inferring insufficient oxygen availability, possibly due to low oxygen solubility in the TZN glass melt.

For the controlled gold crucible corrosion technique, the incorporation of Au^+^ ions into the glass melt also occurs via the oxidation of Au^0^. However, the higher amount of Au^+^ ions in TZN glass suggests that, for the controlled gold crucible corrosion technique, the amount of oxygen available for the oxidation of Au^0^ is higher compared to the gold salt doping technique. Accordingly, we propose that the Au oxidation proceeds at the gold crucible-air-TZN melt interface as there is a virtually unlimited amount of oxygen available in the air atmosphere above the glass melt. Due to thermal convection, purposeful swirling and the low viscosity of the TZN glass melt, the Au^+^ ions formed at this interface are readily dispersed in the glass melt, allowing for further corrosion at the interface. The larger oxygen availability for Au oxidation for the controlled gold crucible corrosion technique due to utilising the oxygen in the air melting atmosphere explains the higher amount of dissolved Au in the glass compared to the gold salt doping technique, having lower oxygen availability due to utilising the limited amount of oxygen dissolved in the melt.

The observed increase of gold crucible corrosion and hence higher amount of dissolved Au with increasing melting temperature can be explained by three possible reasons; (1) reduced viscosity and hence enhanced diffusion, (2) increased oxidation reaction rate and (3) enhanced Au^+^ ion solubility.

**Fig. 2:**
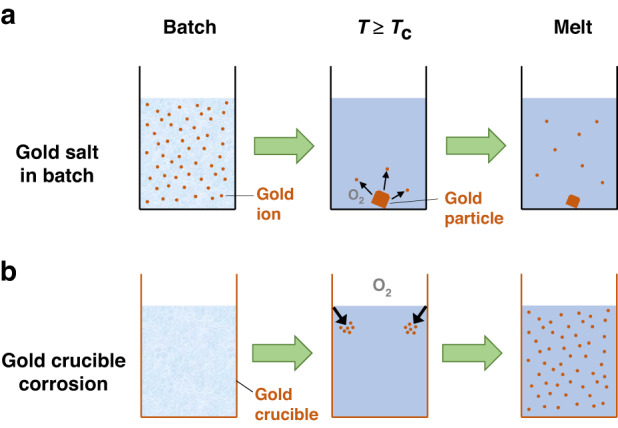
Schematic of the mechanism of the formation of Au^+^ ions dissolved in the glass melt for **a** the gold salt doping and **b** the controlled crucible corrosion techniques, whereby “T” refers to melting temperature and “T_c_” refers to the critical decomposition temperature. The font size of “O_2_” reflects the available amount of oxygen

The different mechanisms of Au dissolution for the gold salt doping and controlled gold crucible corrosion techniques are summarised schematically in Fig. 2 and as follows. For the gold salt doping technique, as the batch temperature increases, first the Au^+^ ions in the gold salt are thermally decomposed to Au metal particles, which are then oxidised above the critical decomposition temperature. The amount of Au^+^ ions in the final glass melt is limited by the size of the Au microparticles formed below the critical decomposition temperatures and the limited amount of oxygen dissolved in the glass melt. For the controlled crucible corrosion technique, the virtually unlimited amount of oxygen in the air atmosphere above the glass melt leads to a higher extent of dissolution of Au from the crucible as Au^+^ ions in the glass melt, whereby the amount of dissolved Au is controlled via melting temperature (and hence melt viscosity, oxidation rate and/or Au^+^ ion solubility) and melting time.

### Transformation of dissolved Au^+^ ions to Au NPs

The second necessary condition for the formation of Au NPs is the reduction of the Au^+^ ions in the glass to Au atoms, which nucleate and grow to Au NPs. For silicate glasses, this is achieved by co-doping polyvalent oxides (such as SnO/SnO_2_) into the glass batch, which act as reducing agents for the Au^+^ ions during reheating of the bulk glass. We tested the viability of this, hereafter referred to as, tin oxide co-doping technique for TZN glass. In addition, we discovered a technique of Au NP formation via reheating powdered starting TZN glass containing only Au^+^ ions but no polyvalent oxides as reducing agents. Note that for both the tin oxide co-doping technique and the glass powder reheating technique, Au^+^ ions were incorporated into the starting glasses through the controlled gold crucible corrosion technique.

#### Tin oxide co-doping technique of Au NP formation

The traditional Au NP formation technique relies on the reducing power of polyvalent elements (PV) that are co-doped into the starting glass together with Au^+^ ions for the controlled formation of Au NPs via reheating the starting glass:

$${{\rm{PV}}}^{x+}+{{\rm{yAu}}}^{+}\to {{\rm{PV}}}^{(x+y)+}+{{\rm{y}}{\rm{Au}}}^{0}$$

where the polyvalent elements are normally selected from Sn, Sb, Pb, etc. that have more than one valence state in glass^7^.

Sn is the most widely used polyvalent element for creating Au NPs in glass, whereby both SnO and SnO_2_ have been co-doped into glass batches at the level of 0.02–2 wt% to generate Sn^2+^ at appropriate concentrations in the glass melt^7,30,31^. Here, we used both SnO and SnO_2_ at 2 wt% doping concentration in the glass batches to investigate the Au NP formation efficacy and effect on the optical properties of TZN glass. The TZN glass batches were melted in a gold crucible at 750 °C for 1 h under air atmosphere, equivalently to the starting glass sample S2 without SnO/SnO_2_ doping.

**Fig. 3:**
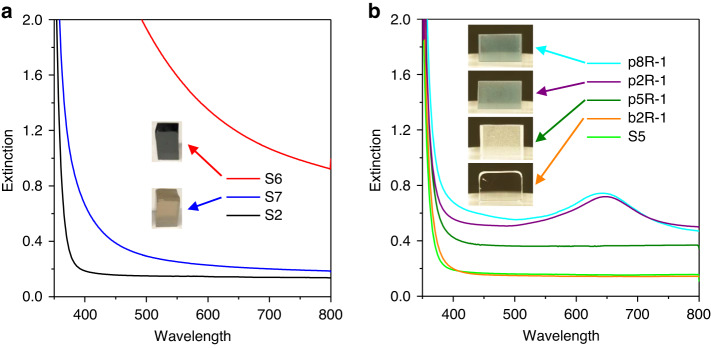
**a** Optical extinction spectra of 2 mm thick glass samples made by melting in gold crucibles: S6 (doped with SnO), S7 (doped with SnO_2_) and S2 (no doping with tin oxide). The inset shows the photographs of S6 and S7. **b** Optical extinction spectra of 2 mm thick starting and reheated glass samples: S5 (batch without Au^+^ ions, melted in a silica crucible), b2R-1 (S2 bulk glass containing Au^+^ ions, reheated in a gold crucible), p5R-1 (S5 powdered glass without Au^+^ ions, reheated in a gold crucible), p2R-1 (S2 powdered glass containing Au^+^ ions, reheated in a gold crucible), and p8R-1 (S8 powdered glass containing Au^+^ ions and doped with ruby (Al_2_O_3_:Cr^3+^) nanocrystals, reheated in a gold crucible). The inset shows the photographs of p8R-1, p2R-1, p5R-1 and b2R-1

As shown in Fig. 3a, both the SnO doped glass sample (S6) and the SnO_2_ doped glass sample (S7) show a distinct colouration, dark brown and light brown colour, respectively. The optical extinction spectra of both samples exhibit a pronounced redshift of the UV edge (more for S6 in agreement with the darker colour) compared to the Sn-free sample (S2) after melting under the same conditions. Similar black/brown coloration and high loss in the visible was observed in an earlier study on TZNL (TeO_2_-ZnO-Na_2_O-La_2_O_3_) based tellurite glass made by adding diamond nanocrystals, which acted as reducing agent, in high concentrations (>20 ppm) to the glass melt at 700 °C^25^, as well as in a multicomponent tellurite glass film prepared under oxygen deficient atmosphere^32^. Such high loss in the visible range was ascribed to the formation of reduced tellurium species (Te^0^ and/or Te^2^^−^). Thus, the brown colour and high extinction magnitude of the S6 and S7 glass samples in this work are also assigned to the formation of reduced tellurium species.

#### Glass powder reheating technique for Au NP formation

The glass powder doping technique has been used to incorporate NPs in phosphate glasses^1^. The new technique of Au NP formation in this work was discovered when investigating the suitability of the glass powder doping technique for homogeneous nanocrystal dispersion in tellurite glass. For this study, to avoid the formation of Au NPs, we selected ruby (Al_2_O_3_:Cr^3+^) nanocrystals as they are chemically non-reducing unlike diamond nanocrystals which have been found to be chemically reducing. First, bulk tellurite glass (S8) was made by melting the batch (without ruby nanocrystals) in a gold crucible. Second, the bulk glass was powdered into a fine powder which was subsequently well mixed with ruby nanocrystals to obtain a well homogenised mixture of glass powder and ruby nanocrystals. Finally, this mixture was reheated at low temperature and short time (570 °C for 10 min) to avoid ruby nanocrystal dissolution, and then cast into a preheated mould. Surprisingly, the ruby nanocrystal doped reheated glass sample (p8R-1) shows a similar blue colour as well as a prominent extinction band in the spectral range of 500–800 nm (Fig. 3b) as diamond nanocrystal doped TZN glass^25^, indicating formation of Au NPs in the ruby nanocrystal doped TZN glass despite the non-reducing nature of ruby nanocrystals. This triggered the hypothesis that the formation of Au NPs in the ruby nanocrystal doped glass was not related to the added ruby nanocrystals but to the fabrication process and the glass itself.

To validate this hypothesis, the glass powder reheating process was repeated at the same heating conditions (570 °C for 10 min) but without mixing ruby nanocrystals into the glass powder. An almost identical homogeneously blue coloured glass sample (p2R-1) was produced as shown in Fig. 3b, indicating Au NPs were successfully created in TZN glass without the use of tin oxide as reducing agent, i.e., only by reheating the TZN glass powders containing Au^+^ ions.

**Fig. 4:**
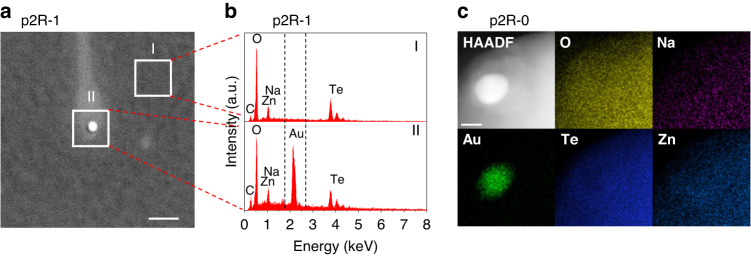
**a** SEM image (backscattering mode) of a 61 nm Au NP near the fractured surface of p2R-1 sample. The scale bar is 200 nm. **b** EDS spectra of region I and II as depicted in (**a**), revealing the elemental constitution of these two regions. **c** HAADF-STEM image with corresponding EDS elemental maps of a 36 nm Au NP found in p2R-0 sample. The scale bar is 20 nm

The existence of Au NPs in the glass samples made by the glass powder reheating technique was proven via electron microscopy (EM) for p2R-1 and also p2R-0, which were made to study the impact of starting glass melting temperature and time (Table 2). The two glass samples were made using reheating for 10 min at 510 °C (p2R-0) and 570 °C (p2R-1). For sample p2R-1, scanning electron microscopy (SEM) imaging in backscattering mode shows a number of bright particles, an example of a particle of 61 nm diameter is shown in Fig. 4a. The energy-dispersive X-ray Spectroscopy (EDS) spectrum for the bright particle (Fig. 4b) exhibits an intense gold signal, while the spectrum from the grey background only shows the elements O, Na, Zn, and Te, which constitute the glass matrix. For p2R-0, scanning transmission electron microscopy (STEM) imaging shows a bright particle of 36 nm diameter, and corresponding EDS elemental maps reveals that this particle is composed of Au, whereas the surrounding matrix contains the glass elements O, Na, Zn, and Te but no Au (Fig. 4c). These EM results confirm that under both reheating conditions Au NPs are formed from the ground glass powder, without adding tin oxide as reducing agent.

**Table 2:** Reheating conditions and Au NP-related properties for reheated TZN glass samples

Sample	T_2_ (^o^C)	t_2_ (min)	LSPR peak (nm)	*D*_*EM*_ (nm)	*D*_*Mie*_ (nm)	*N*_*EM*_ (10^10^/cm^3^)	*N*_*Mie*_ (10^10^/cm^3^)	*N’*_*OM*_ (10^7^/cm^2^)
b2R-1	570	10	n/a	n/a	n/a	n/a	n/a	n/a
p2R-0	510	10	605	33 ± 13	36	6.88 ± 1.53	4.97	n/m
p2R-1	570	10	645	62 ± 9	62	1.23 ± 0.27	0.91	0.39
p2R-2	590	10	680	71 ± 12	78	0.83 ± 0.18	0.56	0.26
p2R-3	610	10	710	84 ± 12	90	0.52 ± 0.12	0.41	0.19
p2R-4	570	20	660	65 ± 10	69	1.07 ± 0.24	0.54	0.28
p2R-5	570	30	698	89 ± 28	86	0.35 ± 0.08	0.19	0.16
p3R-1	570	10	642	60 ± 11	60	4.29 ± 0.70	2.77	1.09
p4R-1	570	10	638	75 ± 23	58	7.21 ± 0.34	4.56	1.65
p5R-1	570	10	n/a	n/a	n/a	n/a	n/a	n/a
p8R-1	570	10	642	n/m	n/m	n/m	n/m	n/m

For the sample name, the first number refers to the starting glass used for reheating, while the second number represents the reheating temperature and time schedule used to make the sample. For p8R-1, the starting glass powder was mixed with ruby nanocrystals prior to reheating. This sample was initially fabricated for another purpose, but it also led to the discovery of a new method for the in situ formation of Au NPs in TZN glass as detailed in the “Results” section. LSPR peak refers to the measured LSPR peak wavelength. *D*_*EM*_ and *D*_*Mie*_ refer to the Au NP diameter determined using electron microscopy and Mie simulation, respectively. *N*_*EM*_ and *N*_*Mie*_ refer to the particles’ volume number density determined using electron microscopy and Mie simulation, respectively. ${{N\text{'}}}_{{OM}}$ refers to the particles’ planar number density determined using dark-field optical microscopy. n/a refers to not applicable due to lack of Au NPs. n/m refers to not measured

#### Glass powder reheating technique—control experiments

To further validate the hypothesis that the powder reheating process itself causes the Au NP formation, we conducted a few control experiments. We commenced with making a control glass sample where a *bulk* glass piece instead of *powdered* glass was used for reheating. Specifically, using the same starting glass S2, the control sample b2R-1 was made by repeating the reheating process (570 °C for 10 min in air atmosphere) used for p2R-1 (showing Au NPs). As expected, the bulk reheated glass sample b2R-1 has the same colourless appearance and absence of any extinction peak in the visible range as the starting glass sample S2 (Fig. 3b). The lack of Au NPs in the bulk reheated glass reveals that Au NPs cannot be effectively created in the entire glass volume by reheating Au^+^ ion containing glass in bulk form. Considering that at 570 °C the low viscosity of TZN glass enables it to flow easily, the absence of Au NPs in b2R-1 cannot be due to the inefficient diffusion of Au atoms (if they exist) to form Au NPs. That is, the lack of Au NPs in b2R-1 is related to the lack of reducing power for the reduction of Au^+^ ions during reheating the bulk piece of S2 glass. This behaviour agrees with previous reports noting the absence of Au NPs formation in silicate glasses containing Au^+^ ions (but no polyvalent ions as reducing agents) after reheating these glasses (in bulk form) in (non-reducing) air atmosphere (e.g., by Stookey^7^, Qiu et al.^33^, and more recently by Kracker, et al.^34^ and Thieme, et al.^35^). All these results indicate that bulk glass itself does not exhibit the ability to chemically reduce Au^+^ ions to Au^0^ atoms when reheated in non-reducing atmosphere.

By contrast, the act of milling Au^+^ ion containing TZN starting glass seems to provide sufficient reducing power for the reduction of Au^+^ ions to Au^0^ atoms, which further nucleate and grow into Au NPs during reheating of the glass powders. As the melting of TZN glass in a gold crucible leads to the incorporation of Au^+^ ions into the glass, it is conceivable that the reheating of glass powder in a gold crucible at a lower temperature may lead to further gold crucible corrosion in such a way that Au NPs are formed immediately from the dissolved Au^+^ ions. To exclude such a possibility, S5 glass (undoped TZN starting glass melted in a silica crucible and thus without Au^+^ ions) was powdered and then heated in a gold crucible under the same reheating conditions as used for p2R-1. The resulting glass sample p5R-1 is colourless (though strictly speaking it is white) and has no extinction band in the visible, indicating the absence of Au NPs in this glass (Fig. 3b). The elevated background extinction generating the white appearance of the sample originates from the light scattering of air bubbles in the glass due to incomplete fusion of the glass particles as also observed for other powder reheated glasses. As for the corresponding starting glass sample S5, there was no Au measured above the solution ICP-MS detection limit of 0.1 ppm in p5R-1. This result reveals inefficient Au dissolution into TZN glass at temperatures <600 °C, at least for the 10 min duration used, which confirms that the powderizing and subsequent reheating of Au^+^ ion containing TZN glass provides the necessary condition for the reduction of Au^+^ ions to Au^0^, and hence allows for the formation of Au NPs that exhibit LSPR. Investigations to reveal the underlying mechanisms for this peculiar phenomenon are still in progress.

### Impact of controlled gold crucible corrosion and glass powder reheating conditions on Au NP size, size distribution and number density

The ability to control the LSPR features of band location, width and intensity is of paramount importance for applications such as enhancing rare earth fluorescence through plasmonic interaction. These features are determined by Au NP size, size distribution and number density. Here, we investigate the impact of dissolved Au content (via gold crucible corrosion during batch melting at different temperatures T_1_) as well as glass powder reheating temperature and duration (T_2_ and t_2_) on the Au NP features of the glass samples.

**Fig. 5:**
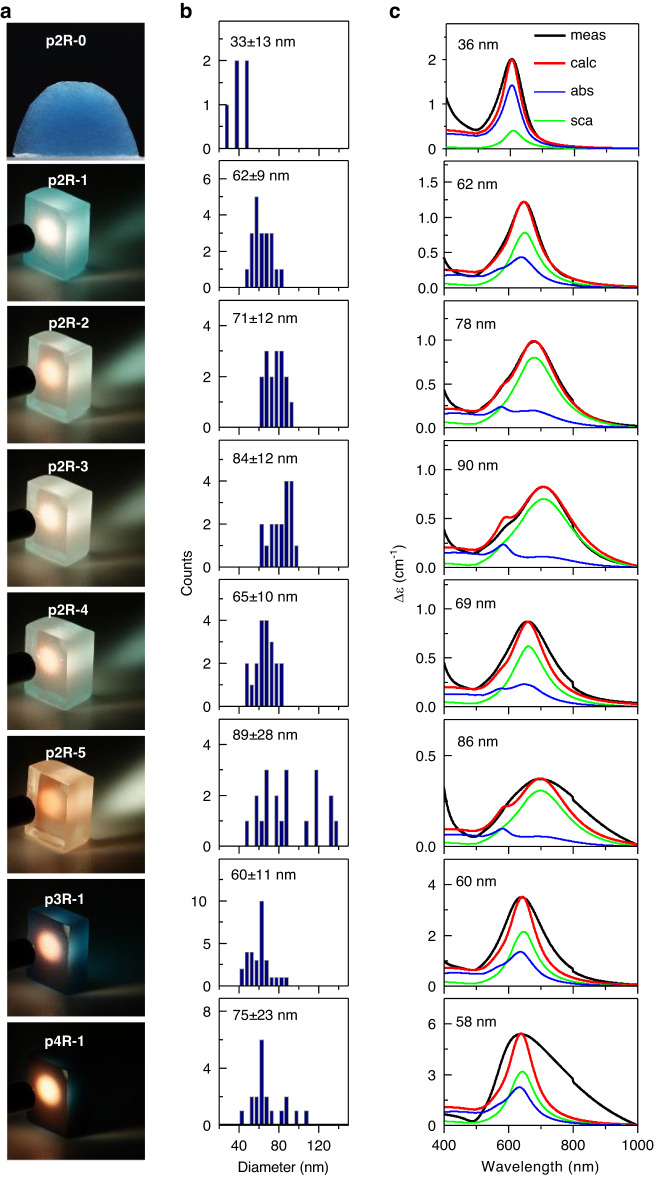
Properties of samples p2R-§ (~6 ppm Au), p3R-1 (~19 ppm Au) and p4R-1 (~75 ppm Au): **a** Photographs showing blue colour of sample p2R-0 and dichroic colour features in p2R-1 to p2R-5, p3R-1 and p4R-1 samples (all with thickness around 5 mm)—transmission of blue light (at the backside of samples) and reflection of orange light (at the front surface of samples), when illuminated with a tungsten filament white light source. **b** Au NP size distributions determined via EM image analysis. The values of each histogram (i.e., xx±xx nm) indicate the average Au NP diameter with standard deviation. Note the different scale for the vertical axis of counts for the different samples. **c** The measured extinction coefficient spectra $\Delta {\varepsilon }_{{meas}}$ (meas, black lines) and the calculated extinction coefficient spectra $\Delta {\varepsilon }_{{calc}}$ (calc, red lines) are overlaid for comparison. For each calculated extinction coefficient spectrum $\Delta {\varepsilon }_{{calc}}$ (calc, red lines), the contributions of the calculated absorption spectrum (abs, green lines) and the calculated scattering spectrum (sca, blue lines) are shown

The photographs of the samples (Fig. 5a) show that they all exhibit a more or less pronounced blue colour in transmission and orange colour in reflection, except for p2R-0 which did not show the reflection feature. The colour and dichroic features indicate Au NP formation in all samples. The difference in transmission and reflection behaviour demonstrates the formation of different-sized Au NPs in the samples.

The Au NP size in the various powder reheated glass samples (listed in Table 2) was derived using two methods; (1) EM image analysis and (2) Mie theory-based calculations of extinction spectra. The EM image analysis yielded Au NP size in the range of 33–89 nm diameter (Fig. 5b). For the Mie theory-based analysis, calculated extinction coefficient spectra of monodisperse Au NPs were fitted to the peak position and height of the measured spectra as detailed in the “Materials and methods” section. The Au NP diameters with the best match of the peak position are in the range of 36–90 nm (Fig. 5c), which agrees well with the Au NP sizes determined via EM image analysis.

The calculated extinction coefficient spectra allow deconvolution into the LSPR absorption and scattering components (Fig. 5c). The scattering component lies in the orange wavelength region and, for p2R-1 to p2R-5 samples, takes up a considerable proportion of the entire LSPR extinction spectrum. This is consistent with these samples showing a considerable colour-specific scattering of orange light when illuminated with white light (Fig. 5a). It is also consistent with dark-field optical microscopy (Supplementary Fig. S5), where the Au NPs appear as bright orange particles that are dispersed homogeneously, with a dark orange background originating from the scattered light of Au NPs out of the focal plane.

The EM images directly show the Au NP size distribution as detailed in the “Materials and methods” section. For samples p2R-0 to p2R-4 and p3R-1 with low Au concentration (6–19 ppm) and made using short reheating time (10–20 min), a narrow size distribution (±~10 nm) was found. Not surprisingly for these samples, a good agreement between calculated extinction spectra of monodisperse Au NPs with measured extinction spectra is observed as noted above. Accordingly, the size distributions determined via EM image analysis and Mie theory-based analysis show a good agreement as detailed in the Supplementary Information. This demonstrates the suitability of our Mie calculation method for determining the average Au NP size for glasses with a narrow size distribution.

In contrast to the samples with lower Au concentration and made using shorter reheating times, p2R-5 made using long reheating time (30 min) and p4R-1 with the highest Au content (75 ppm) exhibit larger size distributions of ±28 nm and ±23 nm, respectively. The widening of the size distribution in these two samples can be attributed to aggregative NP growth^36–39^, whereby longer reheating time for p2R-5 and higher initial Au NP content (due to higher total Au content) for p4R-1 are the driving factors, respectively. Note that for p4R-1, the larger size distribution explains the larger deviation of the calculated extinction spectrum of monodisperse Au NPs with the measured extinction spectrum. For p2R-5, despite also having a larger size distribution, the good agreement between the calculated extinction spectrum of monodisperse distribution with the measured extinction spectrum of polydisperse distribution is attributed to the more symmetric, flat top distribution, whereas p4R-1 has an asymmetric distribution with a pronounced peak on the smaller NP side.

To gain further insight into the impact of Au NP size distribution shape on the position of the extinction spectra calculated assuming monodisperse distribution, Mie spectra were calculated using the size distribution width values of the EM analysis (details of the calculation are given in the Supplementary Information). Supplementary Fig. S4 shows good agreement of this calculated spectrum with the measured spectrum, which validates the suitability of the Mie theory for the calculation of extinction spectra of Au NPs in glass. It also shows that for p2R-5, the symmetric and flat size distribution leads to good agreement between spectra calculated assuming monodisperse distribution and EM measured distribution. By contrast, the asymmetric distribution with a pronounced peak for p4R-1 leads to a significant difference between spectra calculated assuming monodisperse distribution and EM measured distribution. This result demonstrates that the comparison of calculated spectra (assuming monodisperse size distribution) with measured spectra is suitable to deduce broad Au NP size distribution for asymmetric NP size distributions.

The Au NP number density (listed in Table 2) of the samples was determined using both EM image analysis and Mie theory-based calculations of extinction spectra of monodisperse Au NPs. In addition, dark-field optical microscopy was used to derive the Au NP number density in the focal plane (Table 2). The good agreement between the Au NP number density values for these three methods as shown in Table 2 and the Supplementary Information Section 5 demonstrates the consistency of the measurements and analyses.

The impact of the Au content in the starting glasses and the powder reheating conditions on Au NP size, distribution and number density is summarised below. For the samples p2R-§ with fixed Au content of ~6 ppm, the Au NP size increases and hence the Au NP number density decreases with increasing reheating temperature T_2_ and time t_2_. This is confirmed by dark-field optical microscopy, where an increase in T_2_/t_2_ leads to an increase of particle-to-background contrast, indicating an increased population of larger Au NPs with decreased number density (Supplementary Fig. S5). It is also confirmed by the colour change of the samples: the blue colour in transmission decreases, while the orange colour in reflection intensifies, indicating the formation of larger Au NPs. Enhanced diffusion either due to decreased viscosity (i.e., increased temperature) or prolonged heating (i.e., increased time) enables the growth of larger Au NPs.

For the samples p2R-1, p3R-1 and p4R-2 with increasing Au content and made at fixed reheating temperature and time, the Au NP size is approximately constant, indicating that the reheating conditions determine Au NP size, while the Au content has negligible effect. As expected from the observation of similar Au NP size for fixed reheating temperature and time, an increase in Au content leads to an increase in Au NP number density. This is confirmed by dark-field optical microscopy, where the increase of Au content leads to a higher number density of orange Au NPs accompanied by a brighter orange background (Supplementary Fig. S5). Furthermore, the intensification of both the blue colour in transmission and the orange colour in reflection, i.e., of both absorption and scattering, indicates a larger number of Au NPs of similar size for the glasses made from starting glasses with higher Au^+^ ion concentration and using the same powder reheating conditions.

## Discussions

We investigated the incorporation of Au^+^ ions into tellurite glass via the gold salt doping technique and the controlled gold crucible corrosion technique. For both techniques, the critical step for Au dissolution is the oxidation of Au^0^ to Au^+^. For the gold salt doping technique, the Au^0^ occurs in the form of Au metal particles formed via gold salt decomposition upon batch heating. For the controlled gold crucible corrosion technique, the Au crucible is the source of Au^0^. The different amount of dissolved Au^+^ ions in tellurite glass made under the same melting conditions (temperature and duration) for the two techniques is attributed to different levels of available oxygen for the oxidation of Au^0^. For the gold salt doping technique, only a limited amount of oxygen dissolved in the glass melt is available for oxidation. By contrast, for the controlled gold crucible corrosion technique, a virtually unlimited amount of oxygen in the melting atmosphere leads to enhanced oxidation and hence higher amount of dissolved Au^+^ ions, which can be tuned by controlling the melting temperature.

It is worth noting that gold is the preferred crucible material for the melting of tellurite glasses for photonic applications as gold crucibles show very small dissolution less than 0.01 wt% as confirmed in this work, and the dissolved gold ions do not cause any absorption over the entire transmission window of tellurite glass nor does the small amount of gold ions cause any changes to the thermomechanical properties of the fabricated tellurite glass^25,40^. The preference for gold crucibles for tellurite glass melting presents an advantage for the fabrication of tellurite glasses with the controlled in situ formation of gold NPs for plasmonics-based applications.

We found that the tin oxide co-doping technique—which has been extensively used to create Au NPs in silicate glass—is not suitable for tellurite glass as the incorporation of the polyvalent tin oxides SnO or SnO_2_ leads to the formation of undesired reduced tellurium species (Te^0^ or Te^2^^−^) which give rise to detrimental absorption in the visible that impart the glass with a black/grey colour. Inspired by a serendipitous observation, we discovered that reheating *powdered* Au^+^ ion containing tellurite glass (without SnO or SnO_2_) could effectively create Au NPs homogeneously within the glass matrix while reheating corresponding *bulk* glass did not result in obvious transformation of the Au^+^ ions in the glass into the metallic form. Most importantly, we demonstrated that the formation of Au NPs with tunable size, size distribution and number density could be controlled by the amount of Au^+^ ions introduced into the starting glass using the controlled gold crucible corrosion technique together with the reheating temperature and duration of the powdered starting glass. This allows control of LSPR features in terms of band location and intensity. Specifically, increasing the reheating temperature or prolonging the reheating duration leads to the formation of larger Au NPs with lower number density (considering most if not all Au exists as NPs in the reheated glass), resulting in a redshift of the LSPR band and decrease of the peak intensity. For the scenario of fixed reheating temperature and duration, a higher amount of Au^+^ ions in the starting glass predominantly contributes to the formation of more Au NPs in glass of similar size, giving rise to a more intense LSPR band.

We note that the glass powder reheating technique includes the additional step of milling glass into powder. This step is essential for the control of the Au NP formation in tellurite glass as the tin oxide co-doping technique leads to undesired reduction of Te^4+^, while the glass powderization step provides reducing conditions sufficient for the reduction of Au^+^ but not Te^4+^.

It is worth noting that the major limitation of the current powder reheating technique is the presence of gas bubbles in the tellurite glass samples due to the incomplete elimination of gas species during the reheating of the starting glass powders. As this affects the overall glass quality, the development of strategies to reduce and eliminate gas bubbles trapped in the tellurite glass samples is necessary. For example, the use of vacuum during the sintering of silica glass powders has been shown to effectively eliminate pores in the resulting transparent silica glass^41^.

The ability to tune the LSPR features in tellurite glass paves the way towards plasmonics-based research and applications, such as a comprehensive investigation of plasmonic effects on the luminescence of rare earth ions in tellurite glass.

There are several pathways for further investigations to gain insight into the mechanisms of the Au NP formation in tellurite glass, which opens up enhanced control of the Au NP formation and hence LSPR-related properties. One option is to uncover the effect of other processing parameters on the Au NP size, size distribution and number density in tellurite glass, such as batch melting atmosphere (i.e., oxygen partial pressure) for Au^+^ incorporation and particle size of glass powders for Au NP formation. Understanding the cause for the mild reducing power of glass powders for the reduction of Au^+^ (but not Te^4+^) in tellurite glass via the glass powder reheating technique would open up further avenues for control of Au NP formation. Furthermore, the cause for the one order of magnitude lower amount of dissolved Au in tellurite glass compared to silicate is still to be explored, specifically whether the solubility of gold in glass is connected to the solubility of oxygen.

## Materials and methods

### Sample fabrication

All tellurite glass samples were fabricated using the TZN composition of 75TeO_2_–15ZnO-10Na_2_O (in mol%). Commercially sourced raw materials were used: TeO_2_, ZnO, Na_2_CO_3_, SnO, SnO_2_ and HAuCl_4_ with 99.9% or higher purity. HAuCl_4_ aqueous solution (10 mg/mL) was prepared by dispersing HAuCl_4_ salt into a corresponding amount of ultra-pure water (Millipore Milli-Q lab water system) in a thoroughly cleaned glass vial (with reagent grade 69% HNO_3_ and ultra-pure water), which was stored at low temperature (~4 °C) for later use.

All precursor bulk glass samples (hereafter referred to as “Starting” glass samples, S1–S8) were fabricated by melting the ~100 g TZN glass batches (which were undoped, or doped with SnO or SnO_2_, or doped with gold as HAuCl_4_ aqueous solution) in a gold or silica crucible at temperature T_1_ for a duration of t_1_ in air atmosphere, followed by casting the melt in a brass mould (preheated to 240 °C), which was then annealed at ~T_g_ (293 °C for TZN) for 2 h and slowly cooled down to room temperature. Note that for the glass batch doped with HAuCl_4_ aqueous solution, it was homogenised by manually grinding in an agate mortar with an agate pestle for 10 min in air atmosphere, before melting and the subsequent processes. The dopant type and melting conditions are provided in Table 1.

Powders of the bulk starting glass samples were prepared by manually crushing and grinding in an agate mortar with an agate pestle for 10 min in air atmosphere. All starting glass powders used for reheating have particle sizes mostly in the range of 1–100 µm (Supplementary Fig. S3).

Reheating of starting glasses in bulk or powder form resulted in so-called “bulk Reheated” glass samples (b#R- $\S$) and “powder Reheated” samples (p#R- $\S$), respectively, where # represents the number of the starting glass sample and $\S$ represents a certain reheating temperature and time schedule (Table 2).

The “Reheated” glass samples were fabricated by reheating a bulk piece (~4 cm^3^) or the powder of the corresponding starting glass (~20 g) in a gold crucible at temperature T_2_ for a duration of t_2_ in air atmosphere and subsequent quenching of the melt in a brass mould, which was then annealed at ~T_g_ (293 °C for TZN) for 2 h and slowly cooled down to room temperature.

### Characterisation and calculations

#### Solution inductively coupled plasma mass spectrometry (ICP-MS)

Solution ICP-MS was used to determine the total Au concentration (${w}_{{Au}}$) in the starting glass samples (S1–S5) in ppm-weight. Specifically, for each glass sample, ~0.1 g in pulverised form was digested in 10 mL of aqua regia (37% HCl: 69% HNO_3_ = 3:1, in volume fraction) in a Perfluoroalkoxy alkane vial with cover for 48 h at room temperature. After digestion, the sample solution was further diluted 20 fold with ultra-pure water (Millipore Milli-Q lab water system). The Au standard solutions with concentrations of 500, 200, 100, 50, 20 and 10 µg/L prepared by diluting the 10 mg/L Au stock solution with diluted aqua regia (20 fold by ultra-pure water), together with a blank solution of the diluted aqua regia were used for calibration. The sample solutions, together with the calibration solutions, were analysed by solution ICP-MS on an Agilent 7500cx ICP-MS (Adelaide Microscopy) to quantify the Au concentrations in TZN glass samples.

#### Electron microscopy (EM)

EM imaging via Scanning Transmission Electron Microscopy (STEM) or Scanning Electron Microscopy (SEM) and elemental analysis via Energy-Dispersive X-ray Spectroscopy (EDS) were used to identify and measure the size of Au NPs in glass.

##### STEM and EDS

For the sample p2R-0 with the smallest NP size, STEM coupled with EDS was employed to identify Au NPs, using an ultra-high-resolution, aberration-corrected FEI Titan Themis 80–200 operated at 200 kV (Adelaide Microscopy). The glass piece used for testing was pulverised in an agate mortar (in air atmosphere) in the presence of isopropanol for 30 min. The isopropanol solution containing dispersed glass powders (0.1–10 µm) was drop-cast onto a standard copper grid and dried for further STEM and EDS analysis. STEM images were taken in a high-angle angular dark-field (HAADF) configuration to identify Au NPs in glass matrix by the Z-contrast related to the atomic number Z (i.e., brighter pixel indicates a higher atomic number). STEM-EDS maps were acquired to further confirm the elemental nature of Au NPs.

##### SEM and EDS

For all other samples, SEM coupled with EDS was employed to identify Au NPs, using a FEI Quanta 450 FEG Environmental Scanning Electron Microscope operated at 10 kV (Adelaide Microscopy). Each glass piece used for testing was fractured in air atmosphere and then coated with a thin graphite layer (~15 nm) prior to SEM imaging to minimise charging from electron accumulation on the sample surface. SEM images were taken in backscattering mode to identify Au NPs near the surface of samples by the elemental Z-contrast. EDS spot analysis on different regions of each sample was further conducted to confirm the elemental nature of Au NPs.

##### EM image analysis

For the sample p2R-0 with the smallest Au NP size, the size distribution of Au NPs in the sample could only be determined based on 7 different Au NPs due to the difficulty in finding Au NPs under STEM, leading to large uncertainty in the Au NP size distribution. For the other samples, the size distribution of Au NPs was determined from images showing 20–30 different Au NPs.

The Au NP number density determined via EM image analysis (*N*_*EM*_) was obtained by first measuring the total Au concentration (${w}_{{Au}}$) in the sample under consideration via ICP-MS and the volume averaged Au NP diameter (${D}_{{EM}-{VA}}=\root 3\of{\frac{\mathop{\sum }\nolimits_{i=1}^{n}{D}_{i}^{3}}{n}}$, where ${D}_{i}$ is the measured diameter of the i^th^ Au NP) in the sample via EM image analysis. From these two measured values and assuming that all Au^+^ ions were transformed to Au NPs during the powder reheating, the Au NP number density is calculated using known values for Au density (${\varrho }_{{Au}}$ = 19.32 g/cm^3^), TZN glass density (*ϱ*_*g*_ = 5.15 g/cm^3^ ^26^) via ${N}_{{EM}}=6\cdot {w}_{{Au}}\cdot {\varrho }_{g}/(\pi \cdot {\varrho }_{{Au}}\cdot {D}_{{EM}-{VA}}^{3})$.

#### UV-Vis spectroscopy

For both the starting and the reheated glass samples, the optical extinction spectra ($E=-{\log }_{10}T$, where *T* is transmittance) were measured for polished samples of ~2 mm thickness using Agilent Cary 5000 UV-VIS-NIR spectrophotometer. Note that the step at 800 nm in the extinction spectra of most samples is due to detector changeover.

For the reheated glass samples showing distinct LSPR bands, in order to separate LSPR contribution from all other light-attenuation effects such as Fresnel reflection or scattering by gas bubbles, surface defects and possibly non-metallic crystals of the glass matrix and impurities (hereafter referred to as background contributions), the extinction value at 1000 nm (*E*(1000*nm*)) was used as a measure of these background contributions for all wavelengths. By subtracting a constant background value from the whole measured extinction spectrum across 400–1000 nm, a spectrum ($\Delta E(\lambda )=E\left(\lambda \right)-E(1000\text{nm})$) is obtained which represents the LSPR extinction. By normalising the LSPR extinction spectrum ($\Delta E$) to the sample thickness (*d*), the *measured* extinction coefficient spectrum of LSPR ($\Delta {\varepsilon }_{{meas}}=\Delta E/d$) is obtained. Detailed justification of the background subtraction strategy is provided in the Supplementary Information.

#### Mie theory-based calculations

The calculated LSPR extinction cross-section (${\sigma }_{{calc}}$) spectra were obtained using the MiePlot version 4.6.04 software provided by Philip Laven^42^. Input parameters include particle size and size-dependent complex refractive index of Au NPs, as well as real refractive index of the glass matrix.

The use of appropriate dielectric functions (refractive index) of Au NPs is critical to accurately calculate the extinction spectra of Au NPs. To determine if quantum correction beyond the extended Drude model is required in this work, the extinction spectrum of 20 nm Au NPs (lower size limit for the Drude model) in TZN glass was calculated using the extended Drude model-based refractive index of Au via the program provided in ref. ^43^. The peak position of the calculated extinction cross-spectrum for 20 nm Au NPs in TZN glass is at 590 nm, which is at a shorter wavelength compared to the measured LSPR peak wavelengths for all TZN glass samples in this work. This indicates the size of the Au NPs in all the TZN glass samples is larger than 20 nm and, therefore, the extended Drude model is sufficient to correct the refractive index of Au NPs for calculating the LSPR extinction cross-section.

To allow comparison between calculated and measured LSPR spectra, the same method of background subtraction was used, namely the value of calculated ${\sigma }_{{calc}}$ at 1000 nm was subtracted from the whole calculated ${\sigma }_{{calc}}$ spectrum over 400–1000 nm. In this way, the *calculated* extinction cross-section spectrum $\Delta {\sigma }_{{calc}}$ is obtained, which shows zero intensity at 1000 nm as for the *measured* extinction coefficient spectra $\Delta {\varepsilon }_{{meas}}$.

The Mie calculations were employed to find the $\Delta {\sigma }_{{calc}}$ spectrum that fits best to the $\Delta {\varepsilon }_{{meas}}$ spectrum under consideration, which enabled to predict the Au NP size corresponding to the measured LSPR spectrum, based on the following relationship between extinction *cross-section*, *σ*, (based on natural logarithm) and extinction *coefficient*, *ε*, (based on common logarithm)^44^:

1 $$\varepsilon =\frac{{\sigma \cdot N}_{{Mie}}}{2.303}$$

where ${N}_{{Mie}}$ is the number density of Au NPs. The details of the LSPR spectrum fitting and Au NP size prediction are as follows.

First, the calculated LSPR extinction coefficient spectrum ${\Delta \varepsilon }_{{calc}}$ that fits best to the measured spectrum ${\Delta \varepsilon }_{{meas}}$ was obtained as follows: The $\Delta{\varepsilon}_{{meas}}$ spectrum shows a distinct LSPR peak at a specific wavelength (such as 645 nm for sample p2R-1 shown in Fig. 5b). Mie calculation was done by setting a certain particle size (along with the corrected refractive index of Au and refractive index of TZN glass), which yielded ${\sigma }_{{calc}}$ spectrum and subsequently $\Delta {\sigma }_{{calc}}$ spectrum with its peak wavelength best matching that of the measured peak wavelength (by trial-and-error). Then, the Mie calculation based Au NP number density (*N*_*Mie*_) was determined via:

2 $${N}_{{Mie}}=\frac{2.303(\Delta {\varepsilon }_{{meas}{peak}})}{{\Delta \sigma }_{{calc}{peak}}}$$

where $\Delta {\varepsilon }_{{meas\; peak}}$ is the peak intensity of the $\Delta {\varepsilon }_{{meas}}$ spectrum, and ${\Delta \sigma }_{{calc\; peak}}$ is the peak intensity of the ${\Delta \sigma }_{{calc}}$ spectrum. Finally, the whole ${\Delta \sigma }_{{calc}}$ spectrum was multiplied with ${N}_{{Mie}}$ to obtain the calculated (i.e., fitted) LSPR extinction coefficient ${\Delta \varepsilon }_{{calc}}$ spectrum according to Eq. 1.

For a specific Au NPs size distribution, the calculations were done according to:

3 $${\Delta \varepsilon }_{{calc}}=\frac{\sum {{\Delta \sigma }_{{calc}k}N}_{{Mie}k}}{2.303}$$

where$\,{\Delta \sigma }_{{calc\; k}}$ and ${N}_{{Mie\; k}}$ are the calculated extinction cross section and number density of Au NPs with size range *k*, respectively.

To deconvolute the absorption and scattering contributions of the LSPR extinction of monodisperse Au NPs, the calculated (fitted) LSPR absorption and scattering cross sections ${\Delta \sigma }_{{calc\; abs}}$ and ${\Delta \sigma }_{{calc\; sca}}$ were extracted from the calculated LSPR extinction spectrum $\Delta {\sigma }_{{calc}}$. ${N}_{{Mie}}$ obtained via Eq. 2 was then used to multiply the $\Delta {\sigma }_{{calc\; abs}}$ and $\Delta {\sigma }_{{calc\; sca}}$ spectra to obtain the calculated (i.e., fitted) LSPR absorption and scattering coefficient spectrum ${\Delta \varepsilon }_{{calc\; abs}}$ and ${\triangle \varepsilon }_{{calc\; sca}}$ according to Eq. 1.

Due to the mismatch between the experimentally observed non-monodispersed nature of Au NPs in all samples relative to the theoretically monodispersed Au NPs in the Mie calculations, the *N*_*Mie*_ values calculated according to Eq.1 represent an undervalued Au NP number density. For the comparison of the Au NP number densities obtained via Mie calculation and EM image analysis, the *N*_*Mie*_ values were normalised to the *N*_*EM*_ value of the p2R-1 sample.

#### Dark-field optical microscopy

Dark-field optical microscopy was employed as a non-destructive optical technique to identify Au NPs (>50 nm) showing strong LSPR scattering in TZN glass samples p2R-1–p2R-5, p3R-1 and p4R-1, using an inverted Olympus BX51 microscope equipped with a dry dark-field condenser (U-DCD, N.A. up to 0.80). The scattered light from Au NPs within the focal volume was collected by a MPlanFL N 50x / 0.80 objective (MPlanFL N); and the images were taken using a DP50 digital camera employing a 1/2 inch, 1.5 million pixel CCD, with fixed exposure time of 200 ms.

Dark-field optical microscopy was used as an alternative method to determine a measure of the Au NP number density. Specifically, the planar Au NP number density (${{N\text{'}}}_{{OM}}$) represents the number of Au NPs detected per cm^2^ in the focal plane of a dark-field optical microscope image. To allow comparison of the *N’*_*OM*_ values with the *N*_*EM*_ values, the *N’*_*OM*_ values were normalised to the *N*_*EM*_ value of the p2R-1 sample as done for the *N*_*Mie*_ values.

Note that a similar dark-field optical microscope setting (except using 5x/0.15 and 20x/0.45 objectives, with auto-adjusted exposure time) was employed to demonstrate the size range of the TZN glass powders used for reheating.

### Supplementary information

Supplementary Information
