# Supplementary material for: Controlled formation of gold nanoparticles with tunable plasmonic properties in tellurite glass
Source: Light Sci Appl. 2023 Dec 7;12:293. doi: 10.1038/s41377-023-01324-x (PMC10700336; doi:10.1038/s41377-023-01324-x)
Supplement: Supplementary file 1 — Supplementary Information [file 41377_2023_1324_MOESM1_ESM.docx]

**Supplementary Information for**

**Controlled formation of gold nanoparticles with tunable plasmonic properties in tellurite glass**

Yunle Wei^1*^, Jiangbo Zhao^1,2^, Sindy Fuhrmann^3^, Roman Sajzew^4,5^, Lothar Wondraczek^4,6^, Heike Ebendorff-Heidepriem^1*^

^1^Institute for Photonics and Advanced Sensing, School of Physics, Chemistry and Earth Sciences, The University of Adelaide, Adelaide, SA 5005, Australia.

^2^School of Engineering, University of Hull, Hull HU6 7RX, UK

^3^Institute of Glass Science and Technology, TU Bergakademie Freiberg, 09599 Freiberg, Germany

^4^Otto Schott Institute of Materials Research, University of Jena, 07743 Jena, Germany

^5^Leibniz Institute of Photonic Technology, 07745 Jena, Germany

^6^Center of Energy and Environmental Chemistry, University of Jena, 07743 Jena, Germany

^*^Corresponding authors: Yunle Wei (yunle.wei@adelaide.edu.au); Heike Ebendorff-Heidepriem (heike.ebendorff@adelaide.edu.au)

**1. Justification of the background subtraction strategy for the reheated glass samples showing distinct LSPR extinction bands**

The extinction due to Fresnel reflection $E_{F}={log}_{10}(\frac{n^{2}+1}{2n})$, whereby $n$ is the refractive index of the TZN glass as obtained via the Sellmeier equation ^1^, is almost wavelength-independent across 400 to 1000 nm with only a small increase with decreasing wavelength as shown in Figure S1. For micron-sized (non-absorbing) gas bubbles in glass, their scattering of light is independent of wavelength across 200–1000 nm, i.e., adding a constant value to the extinction spectrum ^2^. Taking the sample p5R-1, which contains considerable amount of micron-sized gas bubbles but no Au NPs as an example shown in Figure S1, its extinction spectrum is almost wavelength independent across 400–1000 nm, whereby the small increase of extinction below 420 nm is caused by the tail of the intrinsic absorption edge of TZN glass.

Figure S1: The measured extinction spectrum of 2 mm thick p5R-1 sample and the calculated Fresnel refection for TZN glass. Note that the step at 800 nm in the extinction spectra of most samples is due to detector changeover.

In order to separate LSPR contribution from all other light-attenuation contributions, the extinction value at 1000 nm was used as a constant that was subtracted from the whole measured extinction spectrum across 350–1000 nm, i.e., removing the Fresnel reflection and gas bubble scattering as well as other contributions which are assumed to be wavelength-independent for simplification. Note that a relatively small contribution from LSPR was also eliminated in this case. The thus obtained spectrum ($\Delta E$) was then normalized to the sample thickness ($d$) to obtain the measured extinction coefficient spectrum after background subtraction ($\Delta\varepsilon_{meas}=\Delta E/d$). This background subtraction strategy is demonstrated in Figure S2 taking p2R-1 as an example.


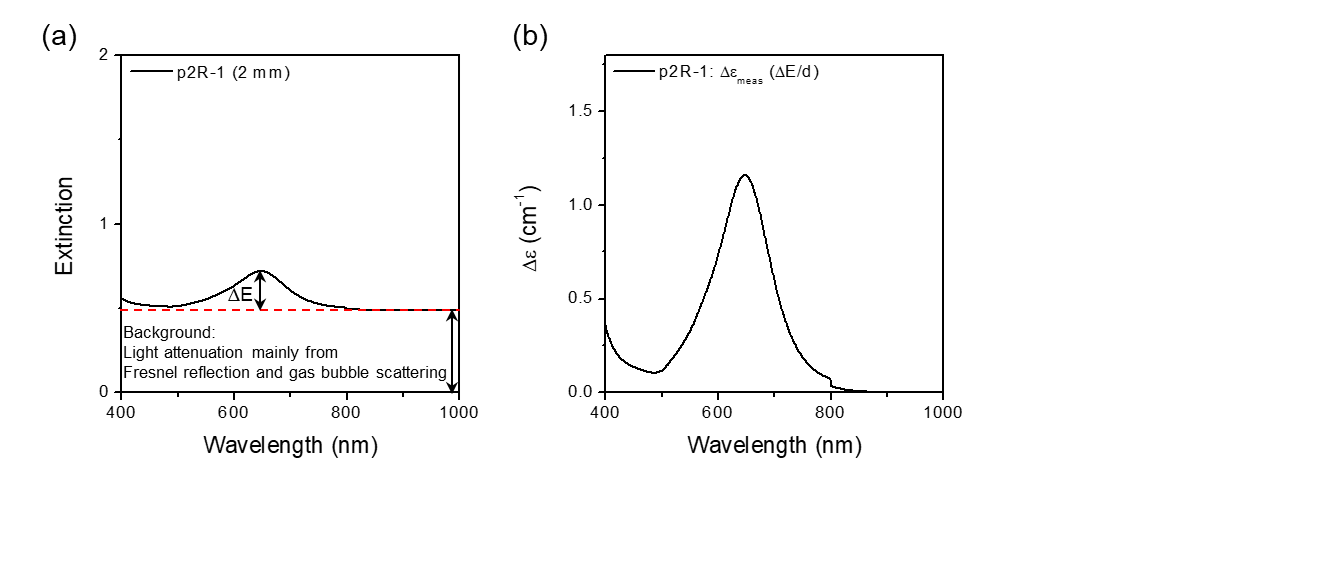


Figure S2: The measured extinction spectrum and performed background subtraction to derive $\Delta E$ (a) and extinction coefficient spectrum $\Delta\varepsilon_{meas}$ ($\Delta E/d$) after background subtraction (b) of p2R-1.

2**. Size range of TZN glass powders**


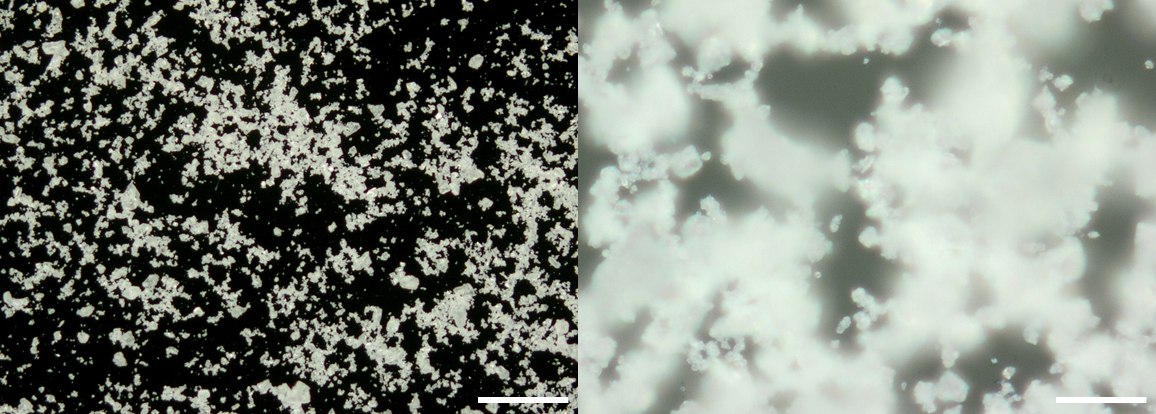


Figure S3: Darkfield optical microscope images showing the size range of TZN glass powders used for reheating. The scale bar is 200 and 20 µm for the left and right images, respectively.

**3. The effect of Au NP size distribution on the LSPR extinction spectra of TZN glass samples containing Au NPs**

Using Equation 3 and the Au NP size distribution measured via SEM (Figure S4a and b), the LSPR extinction coefficient spectra of p2R-5 and p4R-1 samples were calculated and compared with (i) the LSPR spectra calculated via the fitting procedure described above assuming monodisperse Au NP size distribution and (ii) the measured spectra (Figure S4c and d).


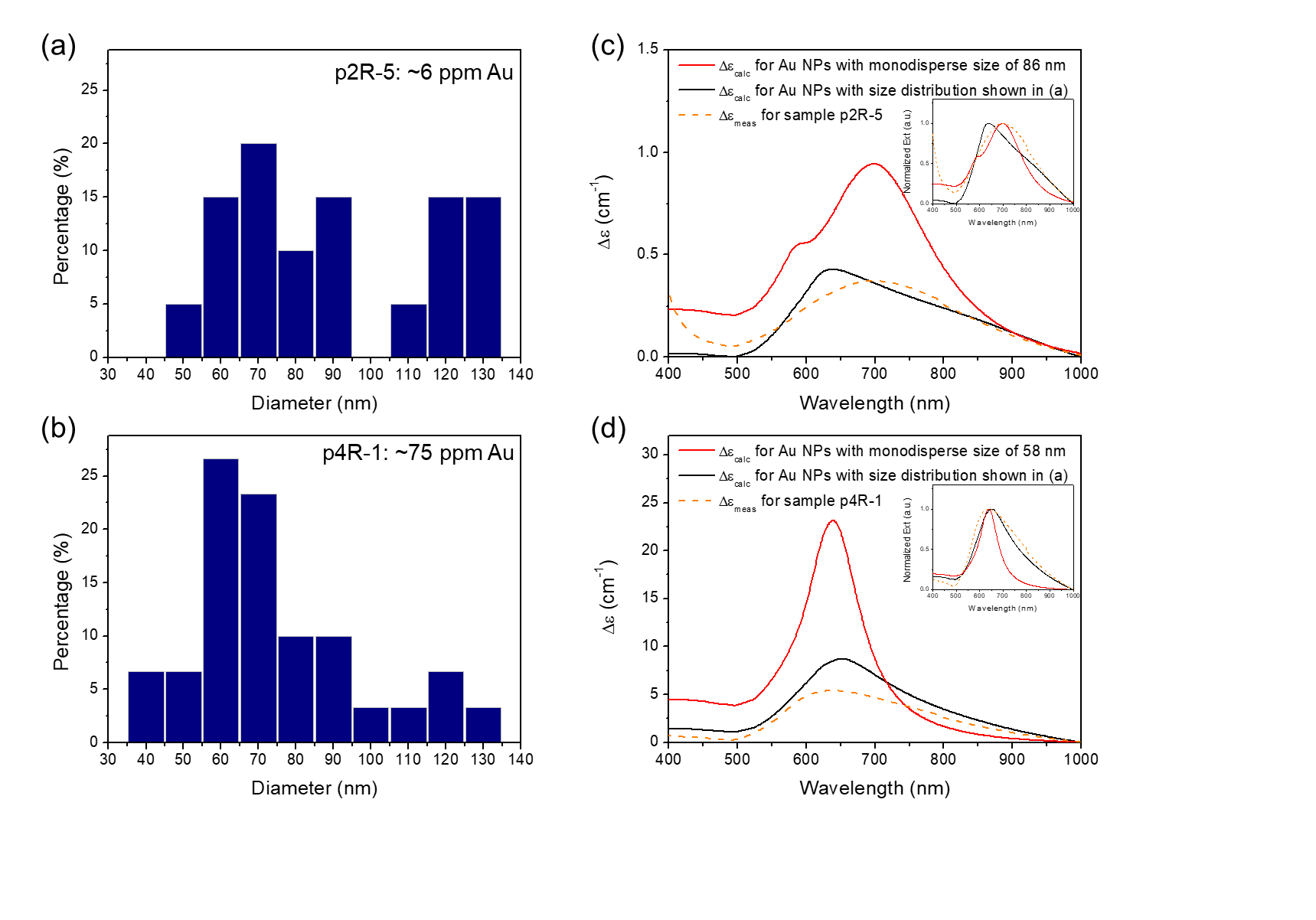


Figure S4: (a) and (b) Au NP size distribution determined via SEM analysis for sample p2R-5 and p4R-1 with ~6 ppm and ~75 ppm Au, respectively. (c) ${\Delta\varepsilon}_{calc}$ for TZN glass containing 6 ppm Au as NPs with monodisperse size of 86 nm or as NPs with size distribution shown in (a), compared to ${\Delta\varepsilon}_{meas}$ of p2R-5 sample containing ~6 ppm Au. Inset of (c) shows the three spectra normalized to their respective peak intensity to give a more direct comparison of their bandwidth. (d) ${\Delta\varepsilon}_{calc}$ for TZN glass containing 75 ppm Au as NPs with monodisperse size of 62 nm or as NPs with size distribution shown in (b), compared to ${\Delta\varepsilon}_{meas}$ of p4R-1 sample containing ~75 ppm Au. Inset of (d) shows the three spectra normalized to their respective peak intensity to give a more direct comparison of their bandwidth.

**4. Dark-field** **optical microscope (OM) images demonstrating Au NPs in TZN glasses**


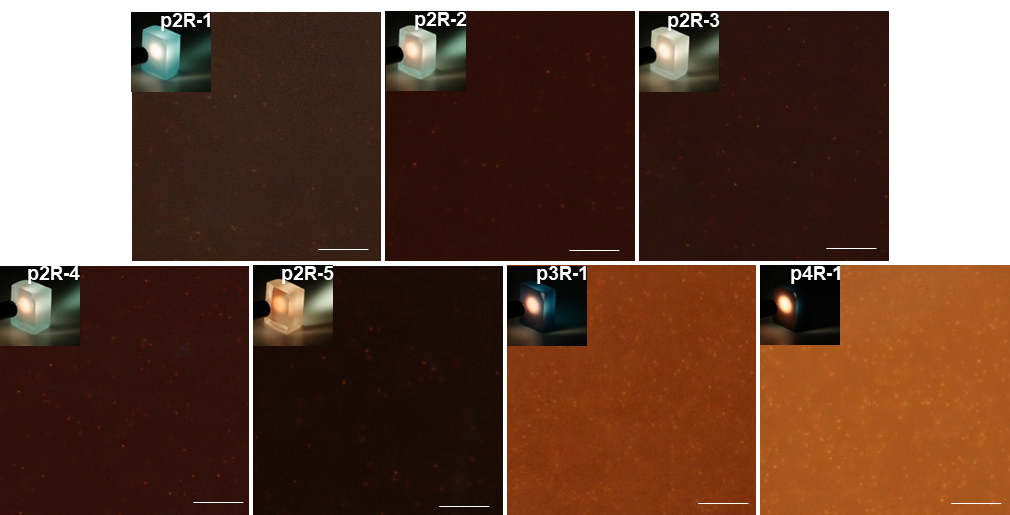


Figure S5: Dark-field OM images showing Au NPs (as bright orange dots) dispersed in TZN glass p2R-1 – p2R-5, p3R-1 and p4R-1, as well as the respective photograph of each sample shown in the inset. The scale bar is 10 μm.

**5. Comparison of the various methods to determine Au NP size and number density**

To evaluate the consistency between the two methods for Au NP size determination, the Au NP size determined via Mie calculation (*D_Mie_*) is plotted as a function of the Au NP size determined via EM image analysis (*D_EM_*) in Figure S6a (values listed in Table 2). For all glasses, the size distribution width of the EM image analysis is within the diagonal where *D_Mie_* equals *D_EM_*. This indicates that *D_Mie_* (determined assuming monodisperse distribution) is within the size distribution width of *D_EM_* (average of measured size distribution). Except for p4R-1 with highest Au content (green data point labelled with ‘75ppm’ in Figure 5d), the *D_Mie_* values are within 10% of the *D_EM_* values (i.e. the data points are within the area bordered by the grey dashed lines in Figure 5d), confirming the narrow size distribution deduced from the good agreement between measured and calculated extinction spectra.

To explore the consistency between the three methods for Au NP number density determination and confirm the conclusions about NP size distribution, the normalized *N_Mie_* and *N’_OM_* values are plotted as a function of the *N_EM_* values in Figure S6b and c, respectively. As for the Au NP size determination, the number density width of the EM image analysis (due to size distribution width) is within the diagonal where *N_Mie_* and *N’_OM_* equals *N_EM_* for all glasses. This indicates that *N_Mie_* (determined assuming monodisperse distribution) and *N’_OM_* are within the number density width of *D_EM_*, indicating the three methods of Au NP number density determination show good agreement in the values. For p2R-0, the slightly lower value of *N_Mie_* relative to the diagonal is attributed to the large measurement uncertainty due to small Au NP size as noted in the main text. For p4R-1, the broad and asymmetric Au NP size distribution leads to a lower value for *N_Mie_*, which was calculated assuming mono-disperse distribution, relative to the diagonal. Regarding the considerably lower *N’_OM_* value relative to the diagonal for p4R-1, the high concentration of Au NPs due to high amount of dissolved Au^+^ ions hampered the identification of single NPs in the OM image analysis, leading to a lower count of Au NPs in the focal plane.


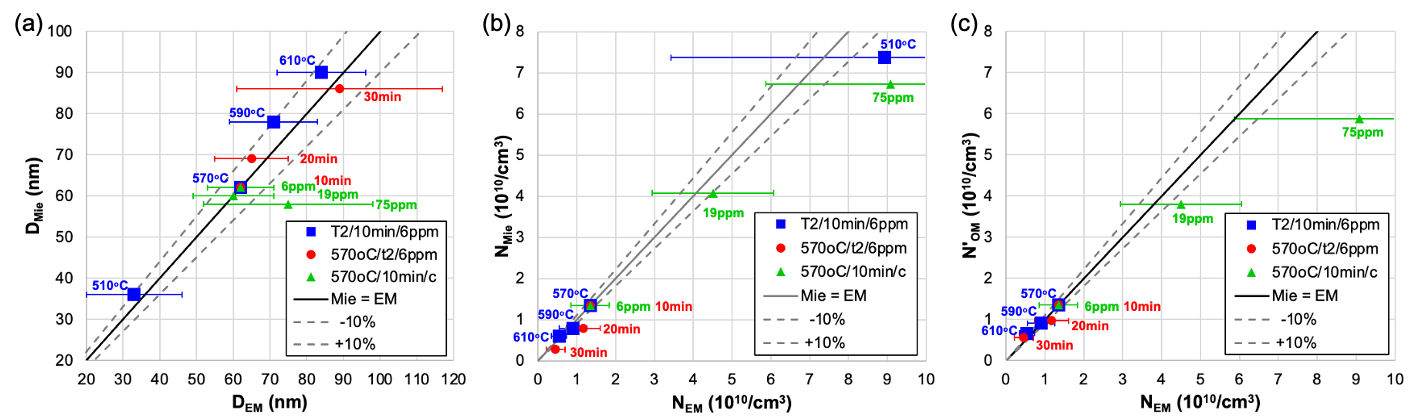


Figure S6: (a) Au NP diameter determined via Mie calculation (*D_Mie_*) as a function of Au NP diameter measured via EM image analysis (*D_EM_*), (b) normalized volume Au NP number density determined via Mie simulation (*N_Mie_*) as a function of Au NP number density measured via EM image analysis (*N_EM_*) and (c) normalized planar Au NP number density determined via OM image analysis (*N’_OM_*) as a function of Au NP number density measured via EM image analysis (*N_EM_*) for p2R-0 to p2R-3 (blue data points, different T_2_), p2R-3 to p2R-5 (red data point, different t_2_) as well as p2R-1, p3R-1 and p4-R-1 (green data points, different gold content). The error bars for *D_EM_* represent the size distribution. The error bars for *N_EM_* are calculated based on the size distribution measured via EM image analysis. The black lines indicate the diagonal where *D_Mie_* equals *D_EM_* in (a) and *N_Mie_* equals *N_EM_* in (b) and *N’_OM_* equals *N_EM_* in (c). The dashed lines indicate the ±10% deviation from the diagonals.

**References:**

1. Zhao, J. B.; Zheng, X. L.; Schartner, E. P.; Ionescu, P.; Zhang, R.; Nguyen, T. L.; Jin, D. Y.; Ebendorff-Heidepriem, H. *‎Adv. Opt. Mater* **2016,** 4, (10), 1507-1517.

2. Sanghera, J. S.; Busse, L. E.; Aggarwal, I. D. *J. Appl. Phys.* **1994,** 75, (10), 4885-4891.
